# Synthesis of Saponite Based Nanocomposites to Improve the Controlled Oral Drug Release of Model Drug Quinine Hydrochloride Dihydrate

**DOI:** 10.3390/ph12030105

**Published:** 2019-07-10

**Authors:** Kumaresan S., Radheshyam Rama Pawar, Bhavesh D. Kevadiya, Hari C. Bajaj

**Affiliations:** 1 Discipline of Inorganic Materials and Catalysis, Central Salt and Marine Chemicals Research Institute (CSMCRI), Council of Scientific and Industrial Research (CSIR), Gijubhai Badheka Marg, Bhavnagar 364002, India; 2 Department of Earth Resources Engineering, Kyushu University, Fukuoka 819-0395, Japan

**Keywords:** quinine hydrochloride dihydrate, controlled drug release, nanocomposite, saponite, oral drug delivery

## Abstract

In the present research study, a 2:1 type of smectite clay minerals, namely natural saponite (NSAP) and synthetic saponite (SSAP), was demonstrated for the first time to be controlled drug release host materials for the model drug quinine hydrochloride dihydrate (QU). The popular sol–gel hydrothermal technique was followed for the synthesis of saponite. The QU was ion exchanged and intercalated into an interlayered gallery of synthetic as well as natural saponite matrices. The developed QU-loaded hybrid composite materials along with the pristine materials were characterized by powder X-ray diffraction (PXRD), Fourier transformed infrared spectroscopy (FTIR), thermal gravimetric analysis (TGA), the Brunauer–Emmett–Teller method (BET) for surface area (SA), and scanning electron microscopy (SEM). The characterization of material results using DSC, FTIR and PXRD confirmed the presence of saponite clay mineral phases in the original and the synthesized saponite samples. Similarly, the drug-loaded composites confirmed the successful intercalation of QU drug on the natural and synthesized saponite matrices. The oral drug release performance of both nanocomposites along with pure quinine drug was monitored in sequential buffer environments at 37 ± 0.5 °C. These composite hybrid materials showed the superior controlled release of QU in gastric fluid (pH = 1.2) and intestinal fluid (pH = 7.4). QU release was best fitted in the Korsmeyer–Peppas kinetic model and demonstrated a diffusion-controlled release from nanocomposite layered materials. The observed controlled drug release results suggest that the applied natural/synthetic saponite matrices have the potential to provide critical design parameters for the development of bioengineered materials for controlled drug release.

## 1. Introduction

The term drug delivery is a pervasive one in pharmaceutical fields, referring to delivering the therapeutic agents in the patient body. Drug delivery systems are different types of routes manipulated for therapeutic effects in our body, such as oral, topical, transmucosal, parenteral, inhalation, etc. However, the oral drug delivery system is one of the most preferred routes, and a convenient option among all type of drugs with a broad range of formulation choices such as tablets, capsules, etc. Usually, conventional drug delivery systems produce unacceptable toxicity, reduced efficiency of drug concentration (more concentration or less concentration), and the therapeutic effect can have some side effects on adsorption sites. In the oral controlled drug delivery system, the amount of drug release would be the same as the standard dosage and at uniform time intervals. By controlling the drug delivery manner, there are more advantages at the action site, such as delivering the drug to the target site, maintenance of drug levels within the desired range, reduction of side effects, and improved patient compliance. Nowadays, better bioavailability, biodegradation, and non-toxic compounds such as polymers, supramolecules, and dendrimers have been employed to improve carriers of drug delivery materials [1,2].

Natural and synthetic clay minerals are important potential materials for material scientists due to their distinctive structural and textural properties and features [3,4]. The clay materials and their composites have been reported over the years in numerous fields of research for several different technological applications [5,6,7,8,9,10,11]. In the biomedical field, the development of nanocomposites was previously extensively used for a wide array of biomedical applications with several bioengineered materials, and controlled drug release approaches in the pharmaceutical industry are considered one of the fastest growing research areas of the past few years [12,13,14]. In controlled drug release system applications, clay–drug nanocomposites are a novel class of composite materials in which clay with excellent nanometer-sized composites is applied as a solid matrix for ion exchanged intercalation of various water-soluble as well as water-insoluble drug molecules [15,16,17]. The nanocomposite should be designed to effectively load the various drug molecules as well as deliver them at the predetermined rate for a predesigned period at a physiological location in the human body. For prevention of the side effects of the toxic drugs, the drug can be delivered to the body in small dosages as well as in controlled release patterns to minimize the side effects and increase patient compliance [18,19].

Recently, our group published several research reports on the different clay-based nanomaterial for drug release applications, where we used different classes and types of drugs [20,21,22,23,24,25,26]. From our past experience, we observed that naturally available montmorillonite [27], hectorite [28], etc., are commonly used as pharmaceutical host materials as the carrier of active ingredients for controlled release of hydrophilic drugs. The abundant natural availability and low cost are major advantages in the application of natural clay minerals. However, the original rigid textural properties along with the presence of impurity phases are the strong limitations in the application of natural clay minerals [29]. Recently we proved that synthetic hectorite clay minerals could be synthesized with high purity phases as well as that the textural properties could be tuned according to the requirements of the application area [21,26,30,31,32]. Saponite clay belongs to the naturally occurring 2:1 trioctahedral layered s phyllosilicate family and is comprised of layers of Si (IV) tetrahedra and Al(III) or Mg(II) octahedra with definite interlayer spacing. The gallery cations can be readily replaced by a variety of functional cations for potential applications in catalysis and adsorption [33]. Considering scarce natural sources, as well the chemical composition sometimes being extremely variable due to the presence of impurities, synthetic analogs of saponite have attracted attention in recent years because of their high purity, adjustable compositions, and superior textural properties [34,35]. We believe that a saponite matrix has unique ion exchange properties that can be used for controlled release of drug molecules by strong electrostatic interactions or hydrogen bonding or dipole interactions with the interlayer gallery of minerals. As per our knowledge, there are still no reports available on the quinine drug release applications of natural and synthetic saponite clay as a host material. Here in this investigation, we are the first to report the interaction of the antimalarial drug quinine hydrochloride dihydride with natural and synthetic saponite clay.

**Figure 1:**
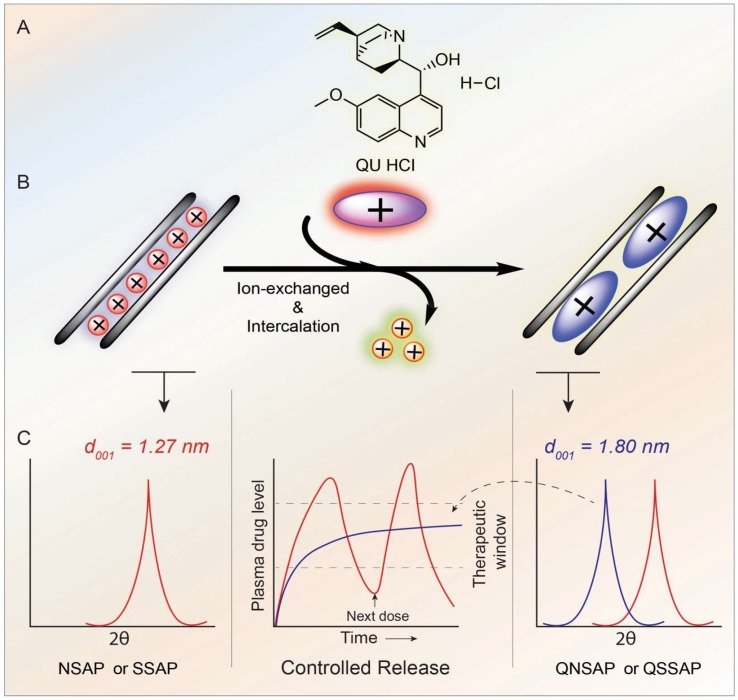
Synthesis and quinine (QU) loaded natural saponite (QNSAP) and quinine (QU) loaded synthetic saponite (QSSAP) for controlled release of QU; (**A**) QU molecular structure, (**B**) QU intercalation by ion exchange in the interlayer gallery of clay by direct insertion or exchange reactions and (**C**) PXRD characterizations of QU from QNSAP and QSSAP nanocomposites and controlled release patterns.

QU is an antiprotozoal and an antimyotonic drug that is known for the treatment of malaria caused by *Plasmodium* species. Controlled release of QU provides a reduction of side effects and increases patient compliance compared to the immediate release of the dosage [26]. The primary objective of this present study was to synthesize saponite smectite clay mineral, its characterizations, and its incorporation of QU molecule. Secondly, the natural saponite–QU nanocomposite was also used to compare with synthetic saponite–QU nanocomposite for controlled release application. The release study of the QU–nanocomposite in physiological conditions demonstrated its controlled release in intestinal pH. The overall scheme of QU–nanocomposite synthesis and QU control release is depicted in Figure 1.

## 2. Material and Methods

### 2.1. Materials

Natural saponite (NSAP) was obtained from the source of clay repository, Clay Minerals Society and used without further modification. Aluminum isopropoxide was purchased from Sigma Aldrich (St. Louis, MO, USA). Quinine hydrochloride dihydrate (QU, C_20_H_24_N_2_O_2_ HCl 2H_2_O) obtained from Sigma Aldrich, USA. Sodium Silicate (Na_2_SiO_2_) Mg (CH_3_COO)_2_, NaOH, KH_2_PO_4_ and HCl were obtained from S.D. fine chemicals (Mumbai, India) and used as received. The cation exchange capacity of the natural Saponite and synthetic Saponite is 76 and 59.7 mequiv/100 g clay, respectively [36,37].

### 2.2. Synthesis of Saponite

Synthetic Saponite clay was prepared by the sol–gel hydrothermal crystallization technique [37]. The gel was formed by mixing [Al(OCH(CH_3_)_2_)_3_], NaOH, Mg (CHCOO)_2_ (with a gel composition of 1SiO_2_, 0.834 Mg (CH_3_COO_2_), 0.113 NaOH, 18.32 H_2_O) in water. The saponite clay with an octahedral sheet consisting of two metal ions incorporated clay was prepared by diluted 40 g of Na_2_SiO_2_ by the addition of 100 mL demineralized water; the 11.9 g of Al [OCH (CH_3_)_2_] solution was dissolved in 80 mL 2M NaOH solution. The aluminum-containing solution was gradually added to Si containing solution under continuous stirring. The gel was formed by allowing it to stand ideal for 1 h. simultaneously, 36 g of Mg (CH_3_COO_2_) dissolved 200 mL of de-ionized water was slowly added into the above Si/Al solution under stirring at 90 °C for 2 h. The gel so formed was aged for 4 h and transferred to the autoclave for hydrothermal treatment at 200 °C for 72 h. The obtained product was filtered and washed with deionized water to remove the unreacted ions. The synthesized saponite is designated as SSAP.

### 2.3. Synthesis of Clay Drug Nanocomposite

The drug-loaded nanocomposite was prepared by adsorption method. One gram of synthetic saponite (SSAP) or/and natural saponite (NSAP) was added to the aqueous QU solution (1 g for QU in 200 mL of water) with continuous stirring (500 rpm) for 24 h at 35 °C. The composite was separated by centrifugation to obtain the hybrid solid residue, dried at 80 °C for 12 h and ground to obtain a fine powder. The obtained QU-loaded nanocomposite was designated as QSSAP and QNSAP for synthetic and natural clay respectively. The drug encapsulation efficiency of clay minerals was calculated spectrophotometrically at λ_max =_ 331 nm using superannuated solution. The amount of QU adsorbed on clay was calculated by the difference in initial concentration and the equilibrium concentration of drug in solution . The QU incorporated clay minerals become hydrophobic in nature.

### 2.4. Characterization

Powder X-ray diffraction (PXRD) analysis was carried out on Rigaku, Miniflex II Desktop diffractometer (Tokyo, Japan) with a scanning rate 3°/min in the 2θ range 2–70°. Fourier transform infrared spectra (FT-IR) were recorded using KBr pellets on Perkin-Elmer GX- FTIR spectrometer. Thermogravimetric analysis was carried out in the temperature range of 50–800 °C at the heating rate of 10 °C/min under a nitrogen atmosphere using Mettler- Toledo TGA/SDTA 851e. The surface area of all samples were determined by N_2_ sorption at 77 K (ASAP 2020, Micrometrics Inc, Norcross, GA, USA) after activating the sample at 423 K under high vacuum. UV-vis absorbance of QU solutions was measured at λ_max_ = 331 nm using UV-visible spectrophotometer (UV-2550, Shimadzu, Japan) equipped with a quartz cell of path length = 1 cm. Separation of all the suspended samples was achieved by Kubota 6500 (Tokyo, Japan) centrifugation instrumentation. Drug release experiments were performed at 35 °C using a Julabo shaking water bath (SW23). FE-SEM analysis was carried out by a JEOL JSM 7100 F instrument. 

### 2.5. In Vitro Drug Release Test

In vitro drug release study of native QU and drug-loaded QNSAP and QSSAP nanocomposite was successfully carried out using the dialysis bag technique in the physiological environment by the pH gradient method . The buffer solution of pH = 1.2 (0.1 M HCl) and phosphate buffer solution pH = 7.4 (4.4 g of Na_2_HPO_4_, 0.38 g of KH_2_PO_4,_ and 16 g of NaCl) was used for the QU release study. The dialysis bags were equilibrated for a few hours with the release medium prepared in 0.1 M EDTA solution. The weighed quantities of nanocomposite (containing approximately 50 mg of QU) were placed in a dialysis bag containing 5 mL of the release medium. The drug release test was carried out using dialysis bag for nanocomposites (QU, QNSAP, and QSSAP) in a shaker water bath at preset temperature (37 ± 5 °C) with 150 mL of dissolution buffer media. The first two hours of the release was carried out in gastric (0.1 M HCl, pH = 1.2) environment and the second phase of the release study was completed in the intestinal pH. After every 30-min time interval, 2 mL of the dissolution medium was collected, which was compensated with the same volume of fresh buffer solution to maintain a constant volume. After 2 h, the release media was changed to phosphate buffer solution (pH = 7.4, 150 mL) and the release study was continued for the next 8 h. Similarly, at 1-h time intervals, 2 mL of the solution was collected and refilled by the same volume of fresh buffer solution of pH 7.4. All the samples were analyzed for QU content by UV–visible spectrophotometer at λ_max_ = 331 nm. These studies were performed in triplicate, and the average values were used in data analysis. To understand the release mechanism of QU from nanocomposites materials, in vitro release data was plotted and fitted in first order, Higuchi, Korsmeyer–Peppas, and parabolic diffusion kinetic models [23].

## 3. Results and Discussion

### 3.1. Powder X-Ray Diffraction Analysis (PXRD)

**Figure 2:**
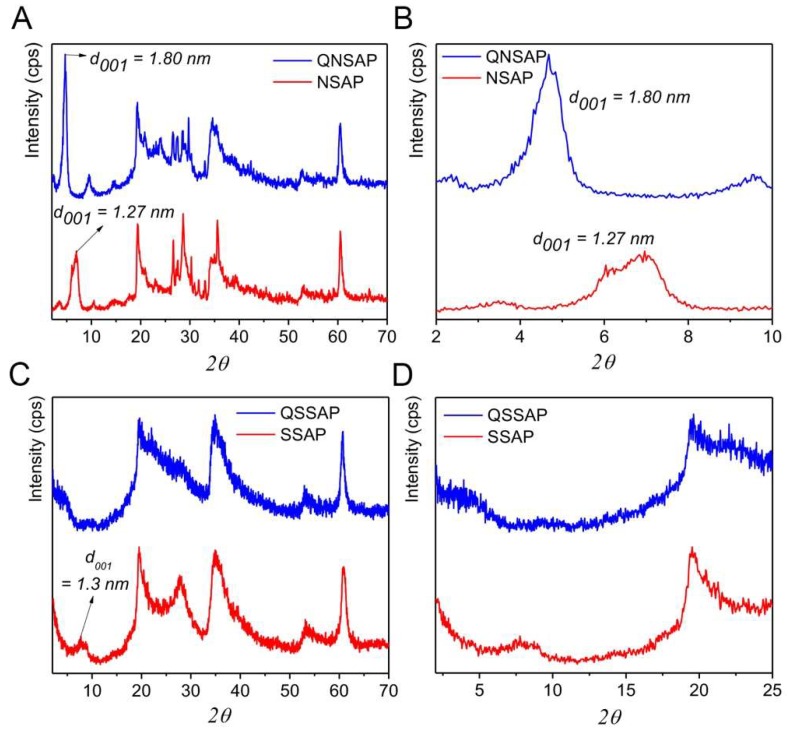
X-ray diffraction patterns of (**A**) NSAP and QNSAP with (**B**) lower angle peaks signature, (**C**) SSAP and QSSAP and (**D**) lower angle scanning plot.

The PXRD patterns of the QU-loaded natural/synthetic nanocomposites are depicted in Figure 2A–D, and Table 1. Generally, 2:1 smectite class of saponite clay minerals show the three characteristic reflection phases in the PXRD patterns at 2θ = 6.8-7-5° (d-spacing 1.25–1.51 nm), 19.4° (d spacing 0.47 nm) and 60.5° (d spacing 0.153 nm) due to (001), (110), and (060) planes typical for the saponite clay. The observed PXRD patterns of the natural/synthetic saponite before drug loading clearly showed three distinct peaks reflection confirming the 2:1 type of saponite clay phases in the original and synthetic saponite samples [34,35]. According to the previous report, first order 001 reflection, basal spacing around 1.515 nm, represent the presence of Ca-saponite, whereas at ~1.25 nm represent the Na-saponite [38]. The natural saponite showed the 001 peaks with a maximum at 2θ = 7.16° with d spacing 1.27 nm, close to the reported Na-saponite. However, for the drug-loaded nanocomposite, PXRD patterns showed the expansion of 001 nanolayer sheets by shifting the 2θ values to 4.77°, indicating the expansion of interlayer distance to 1.8 nm. Additionally, in QNSAP an increase of the intensity and a narrowing of the basal reflection was observed due to a more ordered organization of the layered sheets, occurring in the presence of organic quinine molecules inside the interlayer space [35]. Similarly, synthetic saponite demonstrated a characteristic peak corresponding to 001 planes at 2θ = 6.93°, with an interlayer spacing of 1.3 nm, close to the natural Na-based saponite. After drug loading, QSSAP sample showed complete exfoliation due to high drug loading.

### 3.2. Fourier Transform Infrared Spectroscopy (FTIR)

**Figure 3:**
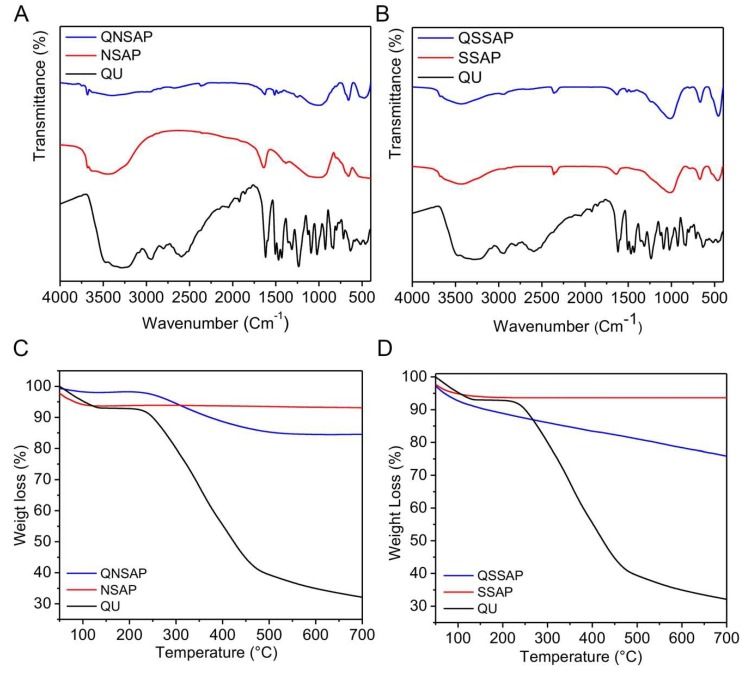
FTIR spectra of (**A**) QU, NSAP, and QNSAP, and (**B**) QU, SSAP and QSSAP. Thermal Gravimetric Analysis (TGA) of (**C**) NSAP, QNSAP, (**D**) SSAP and QSSAP.

Figure 3A,B depicts the FTIR spectrum of QU drug, NSAP, QU-loaded QNSAP nanocomposite and QU, SSAP, and QU-loaded QSSAP nanocomposite respectively. The natural saponite and synthetic saponite depict the absorption bands at 3689/3685 cm^−1^ and 1640 cm^−1^ due to –OH bands of Mg (OH) and octahedral smectite water molecules adsorbed onto the clay surface respectively [8]. The QU-loaded QNSAP, and QSSAP nanocomposite showed the absorption bands at 3679 and 3675 cm^−1^. The absorption band at 3400 cm^−1^ is due to the -OH stretching of both the clay and drug-loaded clay nanocomposite. For QU-loaded natural saponite and synthetic saponite aliphatic (C-H) band vibration are shown at 2340 cm^−1^ and 2949 cm^−1^. The –NH_2_ stretching band of QU-loaded QNSAP and QSSAP are shown at 1639 cm^−1^ and 1638 cm^−1^ respectively. The QNSAP and QSSAP vibration stretching band are observed at 1513 cm^−1^. The FTIR absorption spectra of the clay and drug-loaded clay are shown in Table 2. The functional group identification by the FTIR clearly demonstrates the intercalation of QU in the clay interlayer space. In the previous report, we already confirmed the intercalation of quinine organic moiety on synthetic hectorite clay matrix by solid-state ^13^C MAS-NMR studies [26].

### 3.3. Thermal Gravimetric Analysis (TGA)

The thermal gravimetric analysis (TGA) of clay and drug-loaded clay nanocomposite are shown in Figure 2C,D. The first mass loss below 200 °C in NSAP, SSAP, and nanocomposite QNSAP and QSSAP corresponds to the loss of physiosorbed water. The second weight loss of endothermic peak, observed only in the nanocomposite QNSAP and QSSAP at 200–400 °C, corresponds to the decomposition of quinine moiety [21].

### 3.4. Differential Scanning Calorimetry Analysis (DSC)

**Figure 4:**
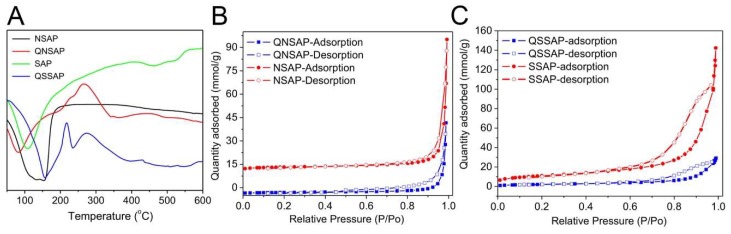
Differential scanning calorimetric analysis of (**A**) NSAP, QNSAP, SSAP, and QSSAP. N_2_ sorption isotherm plots of (**B**) NSAP, QNSAP, and (**C**) SSAP and QSSAP.

The DSC analysis of NSAP, SSAP, and nanocomposite QNSAP and QSSAP is shown in Figure 4A. In NSAP and SSAP, and QNSAP and QSSAP nanocomposite, the first dissociation peak was observed in the endothermic range at 50–180 °C, due to dissociation of the physiosorbed water moiety. The second endothermic peak observed in QNSAP and QSSAP in the range of 180–250 °C in the DSC curve was due to the decomposition of the organic moiety.

### 3.5. BET Surface Area analysis 

**Figure 5:**
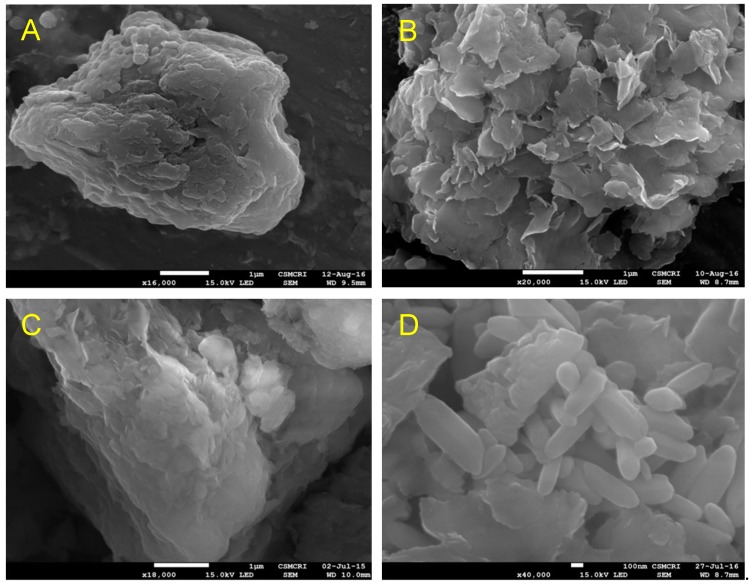
FE-SEM images of (**A**) NSAP, (**B**) QNSAP, (**C**) SSAP, and (**D**) QSSAP.

The N_2_ sorption studies were carried out via analysis by Brunauer–Emmett–Teller (BET) method, to analyze the surface area, pore volume, and pore diameter of NSAP, SSAP, QNSAP, and QSSAP. N_2_ adsorption–desorption data of all the materials are shown in Figure 3B,C and Table 3. The observed results demonstrated that N_2_ isotherm of natural and synthetic saponite represented type IV isotherm nature, according to the International Union of Pure and Applied Chemistry (IUPAC) classification [39]. Additionally, these samples showed H_3_ hysteresis loops. Such hysteresis is usually found in solids consisting of aggregates or agglomerates of particles forming slit-shaped pores, with the non-uniform size or shape, confirming layered clay material . The surface area of the synthetic saponite was 881.66 m^2^/g, close enough to the previously published literature [40]. Further after drug incorporation, the surface area of both NSAP (70 m^2^/g) and SSAP (881.66 m^2^/g) decreased to 36.7 and 181 m^2^/g, respectively. It clearly shows the successful loading of the QU molecule in the clay layer. Similarly, the pore diameter of NSAP (30 nm) and SSAP (15.59 nm) was increased to 33.3 and 18.05 nm, respectively. The increased pore diameter in the drug-loaded samples may be due to the incorporation of organic drug moiety in the clay. These types of pores can be explained as pseudo pores, generally observed in clay samples after organic modification [21]. The surface morphology of NSAP, SSAP with drug incorporated saponite composites QNSAP and QSSAP was studied by SEM (Figure 5A–D). The better compact layered structure was observed in case of the natural/synthetic Saponite SEM images, whereas after drug loading, SEM image showed more dispersed layers structure in both the materials. This is additional evidence for quinine drug incorporation into the interlayer gallery of natural saponite and exfoliated structure of synthetic saponite drug hybrid.

### 3.6. In Vitro Drug Release Study of Clay and Nanocomposite

**Figure 6:**
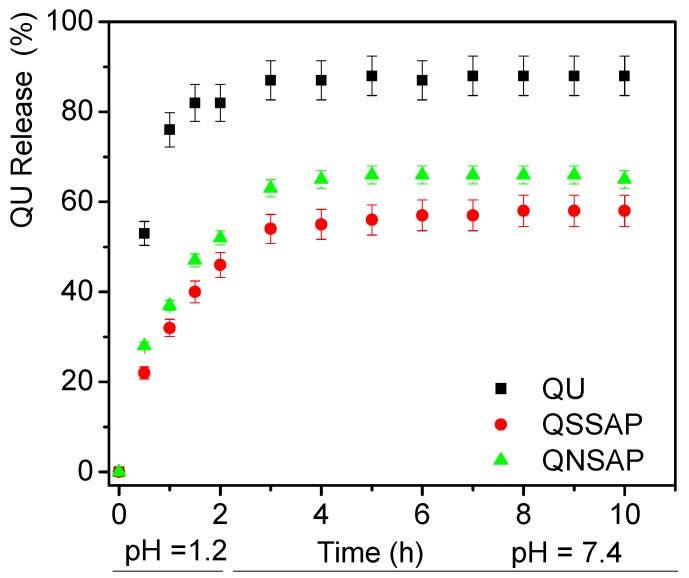
In vitro release of QU from QNSAP and QSSAP nanocomposites and comparison with QU control free drug.

In vitro drug release studies were carried out from the QU, QNSAP and QSSAP natural and synthetic smectite clay–drug nanocomposite at intestinal (pH = 7.4) gastric fluid (pH = 1.2) buffer environment. Total drug loading capacity of nanocomposite is given in Table 4. The controlled drug release of QU and QU-loaded nanocomposite is depicted in Figure 6. At pH = 1.2, 82% QU (without clay composite), 61% quinine from QNSAP nanocomposite, and 40% quinine from QSSAP clay composite were released in two hours. After replacing the release media with a buffer solution of pH = 7.4 (phosphate buffer), 88% QU (without clay composite), 70% QU was release from QNSAP clay drug nanocomposite and 55% from QSSAP over 10 h. Drug release from QSSAP is lower as compared to QNSSAP nanocomposite in both buffer medium. The high surface area, cation exchange capacity, pore volume, higher adsorption capacity and increase in pore size after drug loading in QSSAP may be the reason for controlled release of QU compared to natural saponite and native QU. The release of drug was in the following order QSSAP > QNSAP > QU. 

**Figure 7:**
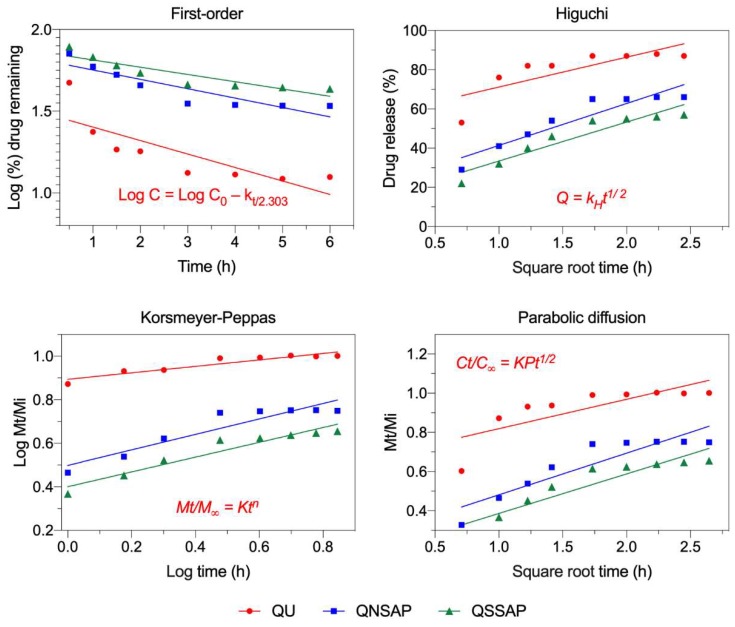
The fitting of QU control release data to first order, Higuchi, Korsmeyer–Peppas and parabolic diffusion kinetic models.

To the assessment of possible QU release mechanism from QNSAP and QSSAP nanocomposite, the release data were fit into an appropriate mathematical model (Figure 7 and Table 5). The release pattern correlated best to the Korsmeyer–Peppas model. According to Korsmeyer–Peppas, the value of n characterizes the release mechanism of the drug as described in Table 1 and the calculated value of n for QU release followed the diffusion controlled mechanism (fickian diffusion) pattern. [41].

## 4. Conclusions

Synthetic saponite was successfully synthesized using the sol–gel hydrothermal method. For the first time, the synthetic saponite was used as a host material for the drug release study with quinine as a guest molecule. We also compared the natural saponite clay with QU nanocomposite and pure QU for controlled release studies. The results confirmed that the synthetic saponite clay provided better-controlled release patterns compared to natural saponite clay and pure QU. The present study also concludes that the synthetic/natural Saponite clay minerals are better materials to serve facile and efficient drug release vehicles.

## Figures and Tables

**Table 1:** The PXRD patterns 2θ valued along with (001) d-spacing of original clay and developed nanocomposites.

Clay and Nano Composites	2θ	001 Basal Spacing (nm)
NSAP	7.06	1.27
QNSAP	4.89	1.8
SSAP	6.93	1.3
QSSAP	-	Exfoliation

**Table 2:** FTIR patterns wavenumber assignment for pristine clay, drug-loaded nanocomposite, and quinine drug.

Sample Type	Assignments	Absorption Bands of NSAP and SSAP, (cm^−1^)	QU (cm^−1^)	QNSAP and QSSAP, (cm^−1^)
Clay	Mg(OH)_2_ stretching -OH (for H_2_O)	3686, 3685 3433, 1640	-	3678, 3675 3432, 1640
	Si-O	1008, 1029	-	1023, 1014
	Si-O-Al Si-O-Mg	680, 669 528, 467	-	667 482, 456
QU	V(C-H) aliphatic	-	2947	2949
	V(Qu-NH_2_ group):	-	1618	1611, 1629
	V(C=C), alkene V(Q ring)	-	1505	1511, 1513
	δ (CH_3_-methoxy)	-	1468 1235	1451, 1467 1260, 1241

**Table 3:** Brunauer–Emmett–Teller (BET) surface area analysis results of original and developed composites samples.

Sample	BET Surface Area, (m^2^/g)	Pore Volume (cm^3^/g)	Pore Size (nm)
NSAP	070.0	0.45	30.0
QNSAP	36.6	0.21	33.3
SSAP	881.0	3.44	15.6
QSSAP	181.0	0.82	18.05

**Table 4:** Quinine drug encapsulation efficiency: results of drug-loaded composite.

Composite	Drug Loading (mg/g)
QNSAP	211
QSSAP	242

**Table 5:** First order, Higuchi, Korsmeyer–Peppas and parabolic diffusion kinetic models fitting parameters were derived from Figure 6. Here linear correlation coefficient (r^2^) and rate constant (K) of the diffusion kinetic models applied to QU release from QNSAP and QSSAP (data considered from first 6 h of release experiments).

Kinetic Models	Parameters	Formulations		
		QU	QNSAP	QSSAP
First order	*r*^2^	0.6642	0.8062	0.8261
	K	−0.16496	−0.11478	−0.08894
Higuchi-equation	*r*^2^	0.6348	0.88	0.8912
	*K_H_*	15.23	21.41	19.88
Korsmeyer–Peppas	*r*^2^	0.8842	0.8772	0.925
	*N*	0.1483	0.3555	0.3399
	*K_HP_*	7.83	3.151	2.51
Parabolic-diffusion	*r*^2^	0.611	0.8318	0.8661
	*Kp*	0.15	0.2126	0.2017
